# Examining and enhancing the reliability of the Arabic version of the Clinical Perfectionism Questionnaire

**DOI:** 10.1186/s43045-022-00261-6

**Published:** 2022-11-22

**Authors:** Mohsen Alyami, Christian U. Krägeloh, Lma Al-Amri, Marcus A. Henning, Hussain Alyami, Dalal Alghamdi, Reuoof Almutari, Oleg N. Medvedev

**Affiliations:** 1 Department of Psychological Medicine, Faculty of Medical and Health Sciences, University of Auckland, Auckland, New Zealand; 2 Department of Psychology and Neuroscience, Auckland University of Technology, Auckland, New Zealand; 3 College of Medicine, Taif University, Taif, Saudi Arabia; 4 Centre of Medical Health Science Education, University of Auckland, Auckland, New Zealand; 5 School of Psychology, University of Waikato, Hamilton, New Zealand

**Keywords:** Clinical perfectionism questionnaire, Perfectionism, Rasch analysis, Psychometrics, Translation, Arabic

## Abstract

**Background:**

Clinical perfectionism has been implicated among risks for developing depression, anxiety, and eating disorders. This study aimed to translate the widely used Clinical Perfectionism Questionnaire (CPQ) into Arabic and examine its psychometric properties. A general population sample of 1598 Saudi adults completed an online survey. Respondents were randomly selected to create two separate samples of *n* = 400 each, thus meeting the sample size recommendations for Rasch analysis. We applied the partial credit Rasch model to one independent sample to investigate and improve the psychometric characteristics of the scale and replicated our findings with another independent sample of the equal size.

**Results:**

Minor modifications were required to address local dependency issues and resulted in a good fit of the Arabic CPQ to the unidimensional Rasch model in both samples. The scale demonstrated unidimensionality, invariance across personal factors, and good reliability (PSI = 0.78). As expected, the scale scores were positively associated with depression, anxiety, and disordered eating behaviors.

**Conclusions:**

Overall, the Arabic CPQ demonstrated robust psychometric properties after minor modifications that did not change the original scale format. The utility and accuracy of the Arabic CPQ can be enhanced by converting ordinal scores into interval scale scores using conversion tables presented in this paper.

## Background

Striving for excellence is considered a positive personality trait; however, when this shifts towards dysfunctional perfectionism in achieving such goals, the utility of this trait can lead to multiple psychopathologies [19, 25]. From a cognitive-behavioral therapy lens, clinical perfectionism is described as “the overdependence of self-evaluation on the determined pursuit of personally demanding, self-imposed, standards in at least one highly salient domain, despite adverse consequences” ([30], p. 778). Clinical perfectionism has been identified as a transdiagnostic construct and is implicated in the etiology and maintenance of multiple mental health disorders including eating disorders [12, 19].

Since the 1960s, perfectionism was viewed as a unidimensional construct revolving around the cognitive elements perpetuating perfectionistic behaviors, including irrational beliefs. This trend continued until the 1990s, when the multidimensional perspective of perfectionism was suggested [13, 16]. This likely led to a noticeable growth of perfectionism research and created contention in the literature over whether perfectionism is a unidimensional or a multidimensional construct [13, 17, 31].

Leading cognitive-behavioral therapists in the area of perfectionism critiqued how multidimensional measures of perfectionism were “equated” with the clinical perfectionism construct [30]. This meant that the added dimensions did not assess clinical perfectionism but instead measured constructs that are related to it, namely beliefs about other people’s standards and the perceptions that others have exceedingly high standards for the individual. Other dimensions included “concern over mistakes,” “doubts about actions,” “parental expectations,” and “parental criticism.” Many of these dimensions, although related to clinical perfectionism, are not core dimensions; hence, Shafran et al. [30] proposed the definition mentioned above focusing on the core elements of clinical perfectionism. This, according to Shafran et al. [30], represented a failure to discriminate between clinical perfectionism itself and its associated factors.

Therefore, from a cognitive-behavioral therapy lens, people suffering from clinical perfectionism have a dysfunctional way of assessing themselves: firstly, such self-appraisal is dependent on striving for and achieving a specific goal; secondly, the self-appraisal is domain-specific (e.g., ongoing striving to meet high goals in the domain of academic perfection) [30].

To measure clinical perfectionism, Fairburn et al. [11] constructed the Clinical Perfectionism Questionnaire (CPQ). The CPQ consists of 12 items that assess the current levels of clinical perfectionism and includes items on cognitive, behavioral, and affective components of clinical perfectionism. Considerable evidence supports the reliability and validity of the CPQ both in clinical [10] and non-clinical samples [7, 9, 32, 34]. According to these studies, a two-factor solution of the CPQ has been often identified, representing perfectionistic strivings and perfectionistic concerns. The former is where the individual sets exceedingly high standards of performance for oneself while the latter is where the individual being overly worried about making mistakes or being judged negatively by others [35].

Within the Saudi context, knowledge about how clinical perfectionism could be related to depression, anxiety, and eating disorders is lacking, possibly due to the absence of a reliable and valid measurement tool. The present study aimed to translate the CPQ into Arabic and to examine its psychometric properties using the Rasch methodology, a robust statistical approach increasingly becoming a gold standard in rehabilitation medicine [36]. The CPQ was chosen over the other multidimensional measures due to its appropriate fit within the cognitive-behavioral therapy model, which is considered the gold standard for treating clinical perfectionism [14].

## Methods

### Participants

This study was a part of a wider research project examining the relationships between clinical perfectionism, depression, anxiety, and disordered eating behaviors among Saudi adults from the general population. A total of 1598 participants completed an online survey. Participants were eligible if they were Saudi adults aged ≥ 18 years and Arabic native speakers. The mean age was 27.23 years (SD 9.38), and the majority of the sample (77.2%) were females. Participants were from all 13 main provinces in Saudi Arabia, with the majority from Mecca province (42.9%) followed by Riyadh province (24.3%).

### Design and procedure

A cross-sectional survey was administered anonymously using Google Forms. Participants were recruited from social media (Twitter and Facebook). A social media invitation containing brief information about the study and an anonymized study link was posted on the authors’ social media accounts (MA, LA, HA, DA, and RA). The study was further shared via e-mail with personal and professional contacts. Eligible participants were also encouraged to share the study link with their personal and social networks. Interested participants opened the anonymized study link, which included a participant information sheet, a consent form, and a study survey. All questions were made mandatory, and thus, participants answered all survey questions for their responses to be recorded. Participation was voluntary, and all participants provided electronic informed consent. Participants received no compensation in any form. Data collection occurred between October 2020 and January 2021.

### Measures

Participants provided background information, including age, sex, and region of residence. Depressive and anxiety symptoms and disordered eating behaviors were assessed using the Arabic versions of the Patient Health Questionnaire-9 (PHQ-9), the General Anxiety Disorder-7 (GAD-7), and the Eating Attitude Test-26 (EAT-26), respectively. Higher scores indicated increased symptoms. The Arabic version of the PHQ-9 [2], GAD-7 [1], and the EAT-26 [3] have demonstrated robust psychometric properties. In the current study, Cronbach’s alpha ranged from 0.86 for EAT-26 and 0.89 for PHQ-9 to 0.91 for GAD-7 total scores. The participants also completed the Arabic version of the CPQ, which is described in more detail below.

### Clinical perfectionism

To measure clinical perfectionism, participants completed the Arabic version of the CPQ [11]. The CPQ consists of 12 items and is scored on a 4-point Likert scale ranging from 1 (not at all) to 4 (all the time). A total score was calculated by adding all item scores, with a possible range between 12 and 48, where higher scores indicated greater levels of clinical perfectionism. Participants were given a working definition of perfectionism and asked to respond to the CPQ items accordingly as it relates to their experience over the past 4 weeks. The CPQ has demonstrated good psychometric properties in studies conducted with English-speaking samples [7, 10]. The CPQ has also been translated into other languages and its psychometric properties explored with samples from Germany [29], Sweden [28], Iran [27], Australia [32], and New Zealand [9]. Permission to use and translate the CPQ into Arabic was obtained from Professor Roz Shafran.

### Translation and adaptation

The CPQ items were translated into Arabic using a forward-backward translation protocol [6]. First, the original CPQ items were independently translated by two study authors (a psychiatrist and a medical student) who are fluent in English and Arabic. Second, both translations were compared, and a provisional draft was produced. Third, the provisional draft was forwarded to another researcher (a psychologist), who translated the provisional Arabic version back into English without inspecting the baseline English version. The forward and backward translations were discussed by the translation team, and there were minor variations that were resolved through consensus. All items were deemed culturally appropriate. The final Arabic version of the CPQ was then tested with a small sample of medical students (*n* = 20). As there were no apparent problems, no further changes were deemed necessary.

### Data analyses

IBM SPSS v.27 was applied to conduct descriptive analyses and estimate the reliability and the factor structure of the Arabic CPQ. The Kolmogorov-Smirnov and the Shapiro-Wilk tests (*p* < 0.01) were used to detect deviations from the normal distribution assumptions. Spearman correlation was used to assess the relationships between the study variables to establish convergent validity. From the entire dataset, two equal-sized random subsamples (*n* = 400) were extracted for Rasch analysis. Rasch analysis requires a sample size between 250 and 500 respondents to control for type I errors because chi-square statistics may be inflated with larger samples if using RUMM2030 [15]. Type II errors should be minimized by including a sufficient number of respondents (e.g., 20 per item) necessary for item calibrations. The rationale for using two samples for Rasch analysis was the following: By splitting up the sample into samples A and B, we were able to replicate our first Rasch analysis with a second sample and thus enhance robustness. Random samples were statistically equivalent in terms of demographic factors such as age and sex, as evidenced by the relevant test statistics included in Table 1.

**Table 1:** Characteristics of the whole sample and random subsamples including tests for difference

Demographic variables		Full sample, *n* = 1598	Rasch A, *n =* 400	Rasch B, *n =* 400	Group differences
Gender	Female	1234 (77%)	299	310	*χ*^2^(2) = 1.21, *p=* 0.55
	Male	364 (23%)	101	90	
Age (years)	Mean (SD)	27.23 (9.38)	27.86 (9.95)	27.23 (9.02)	*F*(2395,2) = 0.75, *p=* 0.48

Rasch analyses were performed by utilizing RUMM2030 [5] and followed the procedure recommended by Tennant and Conaghan [36] and reporting guidelines outlined elsewhere [18]. Two polytomous Rasch models were potentially applicable for the Arabic CPQ, including the rating scale model [4] and the partial credit model [22]. These models assume that differences between item response options vary, but the rating scale model also requires that such differences are similar across all scale items. However, the partial credit model rejects the assumption of item uniformity and implies that response option structure varies across individual items [22]. The likelihood-ratio test is used to test the uniformity of thresholds across individual items, and if significant differences are detected, the unrestricted partial credit Rasch model is applied. The rating scale model is used if the likelihood-ratio test is not significant [36].

Rasch analysis starts with evaluating the overall data fit to the Rasch model, followed by screening of individual items and evaluation of correlations between fit residuals of individual items that may impact the overall model fit. The criteria for the Rasch model fit include non-significant interaction between items and a latent trait (e.g., perfectionism) evaluated by the chi-square test (*p* > 0.05), the individual items fit residuals within the range between − 2.50 and + 2.50, the correlations between fit residuals of individual items not exceeding 0.20, and no differential item functioning (DIF) due to personal factors (e.g., age, sex) [8, 36]. We have created three age categories for DIF testing based on the sample distribution, including 18–22 (35%), 23–29 (35%), and 30–80 (30%). The Person Separation Index (PSI) is typically used to assess the reliability in Rasch model analysis, which assesses the ability of a scale to differentiate between individuals with different levels of a latent trait (e.g., perfectionism). PSI was interpreted in a similar way to Cronbach’s alpha, with 0.70 considered as acceptable reliability for the assessment of groups and 0.80 and higher for assessments of individuals.

Rasch analysis involves an iterative process of modifying and testing psychometric properties to achieve an acceptable fit to the Rasch model, which defines the parameters of an interval measure. Earlier Rasch studies had the tendency to remove misfitting items to achieve an acceptable Rasch model fit, which could affect the construct validity of a measure. Therefore, we considered deleting misfitting items as a last resort and used the novel methodology that involved creating super-items by summing locally dependent items scores that minimizes the measurement error of individual items and improves the Rasch model fit [20, 23, 24].

The Rasch methodology employs principal components analysis of the residuals and uses *t*-tests to examine the unidimensionality of the measure [33]. Unidimensionality is supported if less than 5% of the significant *t*-test comparisons between person estimates computed for the group of items loading high on the first principal component of residuals and the group of items that has low loadings. Unidimensionality is also confirmed if the confidence interval (lower bound) computed for significant *t*-tests is below 5%. When expectations of the Rasch model are met, the distribution of the person-item thresholds is examined to evaluate targeting of the perfectionism in the sample by the Arabic CPQ items thresholds. Finally, the ordinal-to-interval transformation table can be developed using Rasch model estimates, which allows for a conversion of ordinal scale scores into interval-level data to improve the accuracy of the measurement. Statistical significance was determined by *p-*value > 0.05.

## Results

The Rasch model requires a unidimensional solution, and all items were included in the Rasch analysis without making assumptions about any multidimensional structure. If the model misfits, analyses are able to explore to what extent sources of misfit are related to local response dependency between items (i.e., method effects) or local trait dependency (i.e., multidimensionality).

A likelihood-ratio test indicated significant differences between category thresholds in reference to individual Arabic CPQ items (*χ*^2^(21) = 110.72, *p* < 0.001), which supported the suitability of the partial credit model for these data. Table 2 shows the overall Rasch model goodness of fit indices including the baseline and best-fit Rasch analyses of the Arabic CPQ. The baseline analysis (A1) displayed the overall poor fit to the Rasch model reflected by a significant interaction between the items and the latent trait (*p* < 0.001), meaning that the scale is not consistent in measuring perfectionism across different trait levels. However, there was evidence for unidimensionality, the sample targeting was almost perfect with mean persons approximating items mean, and reliability was good with a PSI of 0.78. Table 3 shows the Rasch model fit statistics for individual items, including location, fit residual, and chi-square values, and shows three misfitting items (4, 8, and 11) marked by an asterisk. The residual correlations were examined at this stage, because fit to the Rasch model can be obscured by spurious residual correlations between individual items known as local dependency. Residual correlations between several items that exceeded 0.20 indicating local dependency were identified, with misfitting items 4 and 11 playing a central role.

**Table 2:** Rasch model statistics for the CPQ including the baseline and the best fit analyses of sample A (*n* = 400) and replication with sample B (*n* = 400)

Analyses	Person mean		Goodness of fit		PSI	Unidimensionality *t*-tests	
	Value	SD	*χ*^2^ (df)	*p*		%	Lower bound (unidimensional)
Baseline (A1)	− 0.07	0.72	85.61(36)	< 0.001	0.77	6.0	3.9 (yes)
Best fit (A2)	− 0.09	0.73	86.43 (72)	0.118	0.78	3.5	1.4 (yes)
Baseline (B1)	− 0.13	0.73	125.76 (36)	< 0.001	0.78	4.3	2.1 (yes)
Replication (B2)	− 0.18	0.81	189.38 (72)	0.081	0.80	3.8	1.6 (yes)

*SD*, standard deviation; *χ*^*2*^, chi-square; *df*, degree of freedom; *PSI*, Person Separation Index

**Table 3:** Rasch model items fit statistics of the CPQ including locations, fit residuals, and chi-square for the baseline analysis of sample A

No.	Item content	Location	Fit residual	Chi-square
1	“Have you pushed yourself really hard to meet your goals?”	− 0.06	0.01	6.24
2	“Have you tended to focus on what you have achieved, rather than on what you have not achieved?”	0.18	2.47	11.27
3	“Have you been told that your standards are too high?”	0.19	1.89	3.93
4	“Have you felt a failure as a person because you have not succeeded in meeting your goals?”	0.00	3.82^*^	15.56
5	“Have you been afraid that you might not reach your standards?”	− 0.24	1.41	0.94
6	“Have you raised your standards because you thought they were too easy?”	0.24	− 0.10	2.17
7	“Have you judged yourself on the basis of your ability to achieve high standards?”	− 0.20	− 0.94	11.75
8	“Have you done just enough to get by?”	− 0.07	2.79^*^	2.81
9	“Have you repeatedly checked how well you are doing at meeting standards?”	− 0.21	0.90	2.09
10	“Do you think that other people would have thought of you as ‘perfectionist’?”	− 0.09	0.04	8.45
11	“Have you kept trying to meet your standards, even if this has meant that you have missed out on things?”	− 0.11	− 1.90	17.96^*^
12	“Have you avoided any tests of your performance in case you failed?”	0.36	0.54	2.45

^*^Significant (*p* < 0.01, Bonferroni adjusted) misfit to the Rasch model

Creating super-items by aggregating dependent items can reduce measurement error while improving the Rasch model fit [20, 23]. This approach can distinguish between local response dependency (e.g., method effects from similar item wording) and local trait dependency (e.g., dimensionality). Essentially, using aggregated items represents bi-factor modeling used in classical test theory approaches [20, 21]. Based on the observed local dependency for the baseline model, locally dependent items were aggregated into 2 super-items (2 + 5 + 8, 4 + 10 + 11) by accounting for the strength of residual correlations and conceptual appropriateness. After this modification, the best fit to the Rasch model was evident with strict unidimensionality, non-significant interaction between items and latent trait, and good reliability (Table 2, best fit (A2)). No misfitting items, local dependency, or invariance (DIF) were identified by rigorous post hoc testing. We were able successfully replicate these results with sample B, which demonstrated strict unidimensionality and even higher reliability, PSI = 0.80 (Table 2, replication (B2)).

**Fig. 1:**
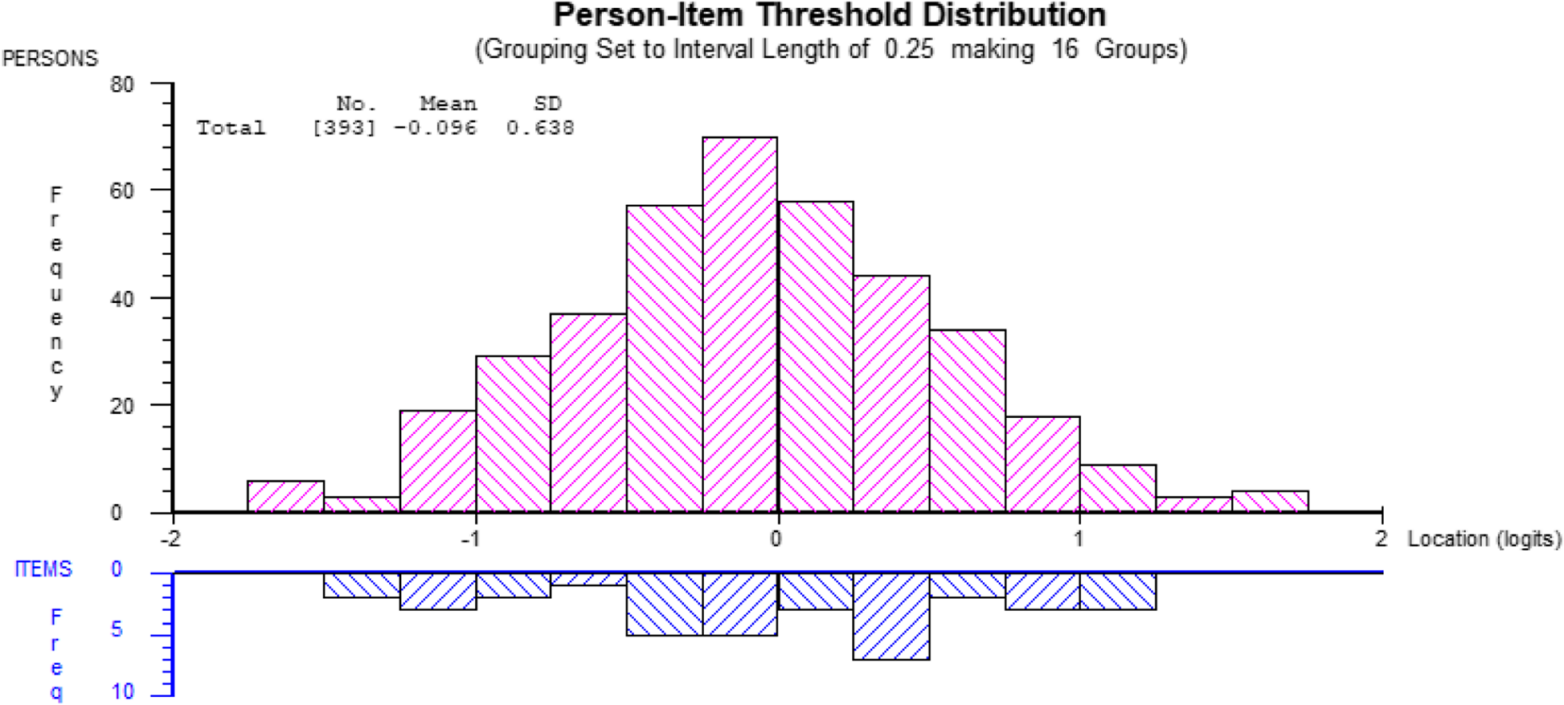
Distribution of person-item thresholds of the best-fit Rasch analysis of the Arabic CPQ (sample A)

Figure 1 shows the distribution of persons and item thresholds derived from the best fit analysis with two super-items. It can be seen that the item thresholds of the modified Arabic CPQ provided excellent sample targeting with no significant ceiling or floor effects, and the sample mean was almost equal to the item mean. This best fit analysis (A2) indicated that after implementing minor super-item modifications, the Arabic CPQ complies with the criteria of the unidimensional Rasch model, which permitted conversion of the ordinal Arabic CPQ scores into interval scale scores using the Rasch model estimates, which is presented in Table 4. This table is user-friendly but requires responses to all items to complete a conversion process, and when total scores are computed, the corresponding interval-level scores can be found on the right-hand side in logit units or in the original scale range for convenience.

**Table 4:** Ordinal-to-interval Rasch conversion scores for the Arabic CPQ in logit units and the original ordinal scale metric

Ordinal scores	Interval scores	
	Logits	Scale
12	− 3.12	12.00
13	− 2.46	15.85
14	− 2.03	18.35
15	− 1.75	19.97
16	− 1.54	21.19
17	− 1.37	22.19
18	− 1.22	23.05
19	− 1.09	23.82
20	− 0.97	24.51
21	− 0.85	25.16
22	− 0.75	25.78
23	− 0.65	26.36
24	− 0.55	26.93
25	− 0.46	27.47
26	− 0.37	28.00
27	− 0.28	28.52
28	− 0.19	29.03
29	− 0.10	29.54
30	− 0.02	30.04
31	0.07	30.53
32	0.16	31.03
33	0.24	31.53
34	0.33	32.04
35	0.42	32.55
36	0.51	33.07
37	0.60	33.61
38	0.69	34.17
39	0.79	34.75
40	0.90	35.37
41	1.02	36.04
42	1.14	36.77
43	1.29	37.60
44	1.45	38.59
45	1.66	39.80
46	1.94	41.43
47	2.38	43.99
48	3.07	48.00

This table should not be used for individuals with one or more missing responses to the scale items

Table 5 presents, for the entire sample, a Spearman correlation matrix for the CPQ and relevant outcome measures: depression (PHQ-9), anxiety (GAD-7), and eating attitudes (EAT-26). CPQ total scores were presented both in the ordinal sum scores as well as those transformed to an interval scale. The correlations for the CPQ total scores with PHQ-9, GAD-7, and EAT-26 total scores were in expected directions and magnitude.

**Table 5:** Spearman correlations between CPQ (ordinal and interval) and the study variables (*n* = 1598)

	CPQ (ordinal)	CPQ (interval)	PHQ-9	GAD-7
CPQ (interval)	0.97**			
PHQ-9	0.24**	0.23**		
GAD-7	0.25**	0.23**	0.79**	
EAT-26	0.11**	0.10**	0.21**	0.23**

*CPQ* Clinical Perfectionism Questionnaire, *PHQ-9* Patient Health Questionnaire, *GAD-7* General Anxiety Disorder-7, *EAT-26* Eating Attitude Test-26

**p < 0.001 (two-tailed)

## Discussion

The current study developed the Arabic version of the CPQ and examined its psychometric properties in a large non-clinical Saudi adult sample using Rasch analysis. Our findings confirmed that the Arabic CPQ, with minor modifications, meets the expectations of a unidimensional Rasch model. Two super-items were created in which locally dependent items were combined to improve the Rasch model fit and reduce measurement error. This methodology has been increasingly used with various scales [23] and was found effective when resolving local dependency between items and related spurious correlations affecting the unidimensionality of scales. After a minor modification, the Arabic CPQ demonstrated unidimensionality, no misfitting items, no local dependency between items, invariance across age and sex variables, and good reliability in both samples A and B (PSI of 0.78 and 0.80, respectively). Correlations between total scores on the CPQ and measures of depression, anxiety, and disorder eating behaviors were positive and significant, similar to previous research [10, 27, 28, 32].

In this study, the creation of super-items solved the local dependency problem that affected the baseline Rasch model fit and unidimensionality of the scale. It should be noted that super-items help to reduce measurement error and spurious correlations for the unidimensional scale only and cannot be generated for a multidimensional scale [26]. Meeting expectations of the unidimensionality, the Rasch model fit accompanied by sound reliability supports the argument that the CPQ represents an adequate measure of an overall clinical perfectionism construct. Based on these results and Rasch model estimates, an ordinal-to-interval conversion table was generated, which enhanced the precision of the CPQ. This ordinal-to-interval conversion table (Table 4) can be used to transform CPQ raw scores into interval-level data to better suit parametric statistical tests and to avoid the violation of their fundamental assumptions [23]. Overall, these findings demonstrated with two independent samples of adequate size that the Arabic CPQ has robust psychometric properties.

Some of the previous research that investigated the factorial structure of the CPQ proposed an overarching construct of clinical perfectionism with two factors [9, 10, 32, 34]. The first factor represents the perfectionistic strivings, and the second factor reflects the perfectionistic concerns. Items included in each of these factors were broadly consistent with other studies. However, evidence for substantial cross-loading of item 7 and low item-total correlation for item 8 [9] may indicate that a two-factor structure may not be very stable. Similarly, in other studies, items 7 and 8 had substantial cross-loadings [34], and items 2 and 8 had low factor loadings and low item-total correlation [32]. For the present Arabic version, a one-factor solution was tenable, particularly after some of the local response dependency had been addressed through subtests. The resulting fit was excellent, and the unidimensional model provides the most parsimonious solution for the Arabic version of the CPQ.

A strength of this study is that Rasch analysis was applied to analyze two large independent samples, which increased the robustness of our findings. An ordinal-to-interval conversion table was produced based on the estimates of the Rasch model to improve the accuracy and precision of the Arabic CPQ and its suitability for parametric statistics. A few limitations need to be noted. First, although the sample size was large (> 1500), our sample was predominantly female (77.2%), which may not be representative of the general Saudi adult population. Also, participants were recruited online from social media platforms using a snowball sampling strategy, which was practical during the COVID-19 pandemic with the restriction measures put in place but may have resulted in a homogenous and biased sample. Future research should aim to achieve better demographic representation. Finally, although the present study provided evidence for the psychometric properties of the Arabic CPQ for Saudi adults from the general public, further data drawing on clinical samples such as OCD and eating disorders are required.

## Conclusions

Accurate assessment of clinical perfectionism is important because it is a risk and maintaining factor for a number of psychological conditions including depression, anxiety, and eating disorders. The current study validated the Arabic CPQ version in a large non-clinical sample of Saudi adults using the modern Rasch methodology. Our results demonstrated that the Arabic CPQ complies with the criteria of fundamental measurement defined by the Rasch model after minor modifications that did not change the original scale format. The ordinal-to-interval conversion tables presented in this paper can be used to further enhance the utility and precision of the Arabic CPQ.

## Data Availability

The datasets generated during and/or analyzed during the current study are available in the [OSF] repository, https://osf.io/b4uyq/?view_only=557604830f7b49fd82e36b64b324bb6d

## Abbreviations

CPQ: Clinical Perfectionism Questionnaire
DIF: Differential item functioning
EAT-26: Eating Attitude Test including 26 items
GAD-7: General Anxiety Disorder scale including 7 items
PHQ-9: Patient Health Questionnaire including 9 items
